# ePWV as a scalable risk factor for large-scale glaucoma screening: evidence from a national Chinese cohort

**DOI:** 10.3389/fcell.2025.1700378

**Published:** 2025-10-27

**Authors:** Yupeng Xu, Zihan Yin, Qiaoyun Gong, Mingjie Zhu, Chuming Lu, Haiyan Wang

**Affiliations:** Department of Ophthalmology, Shanghai General Hospital, Shanghai Jiao Tong University School of Medicine, National Clinical Research Center for Eye Diseases, Shanghai Key Laboratory of Ocular Fundus Diseases, Shanghai Engineering Center for Visual Science and Photomedicine, Shanghai engineering center for precise diagnosis and treatment of eye diseases, Shanghai, China

**Keywords:** glaucoma, AI, estimated pulse wave velocity, incidence, prospective cohort study

## Abstract

**Background:**

To examine the association between estimated pulse wave velocity (ePWV), a marker of arterial stiffness, and glaucoma incidence in Chinese cohort, highlighting ePWV’s potential as a scalable population-level risk factor for glaucoma screening.

**Materials and methods:**

Data were obtained from the China Health and Retirement Longitudinal Study (CHARLS). A total of 11,968 adults aged ≥45 years without glaucoma at baseline (2011–2018) were followed for up to 7 years ePWV was calculated from age and blood pressure and divided into quartiles. Cox proportional hazards models assessed risk, while restricted cubic spline and two-piecewise models explored dose–response patterns. Subgroup analyses tested effect modification by demographic and lifestyle factors.

**Results:**

During a 7-year follow-up, participants in the highest ePWV quartile (≥10.58 m/s) had a higher risk of glaucoma compared with the lowest quartile (<8.01 m/s) (HR [hazard ratio] 1.39, 95% CI [confidence interval] 1.00–1.93). Each 1 m/s increase in ePWV was associated with a 7% higher glaucoma risk (HR 1.07, 95% CI 1.01–1.13). The dose response relationship was linear without evidence of a threshold. Associations were consistent across most subgroups. Sensitivity analyses showed that ePWV was a stronger predictor of glaucoma than age or blood pressure alone.

**Conclusion:**

Higher ePWV independently links to greater glaucoma risk in middle-aged and older Chinese. This observed association indicates that ePWV provides incremental predictive value beyond traditional demographic or clinical factors. Building on this characteristic, incorporating ePWV into future artificial intelligence (AI)-enabled glaucoma screening models may potentially contribute to improving risk-stratification accuracy and facilitating early identification of high-risk individuals.

## Introduction

Glaucoma is one of the world’s leading causes of irreversible blindness. In 2020 an estimated 80 million people were living with glaucoma, making it the second leading cause of blindness worldwide (Tham et al., 2014; Jayaram et al., 2023).Projections from a recent meta-analysis estimate that the number of people aged 40–80 years with glaucoma will rise from 76.0 million in 2020 to 111.8 million by 2040, with Asia and Africa bearing the largest share of cases (Tham et al., 2014; Song et al., 2017). In China, rapid population ageing and urbanization are likely to amplify this trend, posing a major public health challenge (Tang et al., 2019). Glaucoma is asymptomatic in its early stage and silently impairs visual function, typically being identified only when it progresses to an advanced stage. Given that glaucomatous damage is irreversible, early screening and detection of glaucoma are of critical importance. Against the backdrop of China’s substantial population base^4^, the number of glaucoma specialists is vastly inadequate to support large-scale population-wide screening. Thus, the development of artificial intelligence (AI)-enabled systems for glaucoma screening holds vital significance for both clinical practice and public health.

Recent advances in AI have greatly enhanced glaucoma screening, particularly via fundus photography and optical coherence tomography (OCT) scans, enabling automated detection of structural optic nerve changes with high sensitivity and specificity (Dong et al., 2022; Ran et al., 2022; Chen and Bai, 2025; Djulbegovic et al., 2025).However, AI methods based solely on ocular images face limitations: they may underperform in cases with ambiguous disc appearance, myopia, or early vascular pathology.AI models trained on multimodal data, including fundus photographs, OCT scans, and physiological parameters, have demonstrated performance comparable to, or even superior to, human ophthalmologists in detecting early glaucomatous damage (Dong et al., 2022; Wang et al., 2024; Djulbegovic et al., 2025; Koornwinder et al., 2025), and providing appropriate physiological parameters within such AI systems is the foundation for these systems to exert their maximum effectiveness. Therefore, our research focus is precisely on identifying quantifiable, objective, and most importantly glaucoma related physiological parameters that can be effectively integrated into the development and optimization of AI models to enhance their performance in early glaucoma detection and risk stratification (Chen and Bai, 2025; Djulbegovic et al., 2025).The combination of imaging and easily obtained systemic markers presents a promising path toward more robust and scalable AI-based glaucoma screening, particularly in settings where access to ophthalmic specialists or advanced imaging is limited (Chen and Bai, 2025).

In glaucoma pathogenesis, increasing interest lies in the role of vascular dysfunction (Shoshani et al., 2012; Shin et al., 2022), according to the vascular theory of glaucoma that fluctuations in ocular blood flow and impaired autoregulation may damage the optic nerve head (Shoshani et al., 2012). Emerging evidence supports an association between increased arterial stiffness, a prominent indicator of vascular dysfunction, and glaucoma (Lee et al., 2021; Beros et al., 2024). Arterial stiffness is commonly measured by pulse wave velocity (PWV), while in large-scale studies, an estimated PWV (ePWV) derived from age and blood pressure has been introduced as a convenient surrogate for direct PWV measurement, and it has shown strong predictive value for cardiovascular events and mortality (Ohkuma et al., 2017; Lee et al., 2021; Cheng et al., 2024). In a large prospective study conducted in Western populations, higher estimated pulse wave velocity (ePWV) was associated with a significantly increased risk of incident glaucoma (Beros et al., 2024; Yang et al., 2024). These findings suggest that arterial stiffness could serve as a novel marker of glaucoma risk and a potential target for preventive strategies.

Despite this emerging evidence, the relationship between arterial stiffness and glaucoma remains underexplored in Asian populations. We therefore used longitudinal data from the China Health and Retirement Longitudinal Study (CHARLS) (Li et al., 2025), a nationally representative cohort of adults aged 45 years and older, to examine the association between ePWV and incident glaucoma-a parameter whose quantifiable, accessible nature also makes it a promising input for AI-driven glaucoma screening models. Leveraging the large sample size and comprehensive follow-up of CHARLS, we aimed to determine whether higher ePWV predicts future glaucoma beyond established demographic, lifestyle and clinical risk factors, with findings that could further validate ePWV’s utility for integrating into AI-based risk stratification tools. The results of this study may provide new insights into the vascular contribution to glaucoma and inform risk stratification and prevention strategies in ageing populations, including the development of AI screening systems tailored to Asian populations, where such tools are urgently needed to address specialist shortages.

## Materials and methods

### Study design

This was a prospective, population-based cohort study using data from the China Health and Retirement Longitudinal Study (CHARLS), a nationally representative survey of adults aged ≥45 years, conducted from 2011 to 2018. Participants without glaucoma at baseline were followed for up to 7 years to assess the association between estimated pulse wave velocity (ePWV) and incident glaucoma.Patients and the public were not involved in the design, conduct, reporting or dissemination plans of our research.

### Participant selection process and study setting

The CHARLS study employed a stratified, multistage probability sampling design covering 150 counties/districts and 450 villages/urban communities across 28 provinces, thereby ensuring broad demographic and geographic representation. Data were collected using structured household questionnaires, clinical examinations, and blood-based biomarkers. Participants with BMI ≥40 kg/m^2^ were excluded due to the rarity of such cases in our cohort (n = 49, <0.2%) and the increased likelihood of measurement error, which could bias covariate adjustment.

A total of 25,586 participants were recruited. Exclusion criteria included: (1) only one glaucoma survey available (n = 3,398), (2) missing age or blood pressure information (n = 9,495), (3) age <45 years (n = 425), (4) missing key covariates (n = 194) including sex, education, marital status, location, or BMI ≥40, and (5) self-reported glaucoma at baseline (n = 106). After exclusions, 11,968 participants with complete data were included for analysis of the association between ePWV and incident glaucoma.

### Ethical statement

The CHARLS protocol was approved by the Biomedical Ethics Review Committee of Peking University (IRB00001052-11015) and conducted in accordance with the Declaration of Helsinki. Written informed consent was obtained from all participants. This study adheres to the Strengthening the Reporting of Observational Studies in Epidemiology (STROBE) guidelines.

### Assessment of ePWV and covariates

ePWV, a validated non-invasive marker of arterial stiffness, was calculated using the formula developed by Greve et al. (2016), based on age and blood pressure parameters (Greve et al., 2016; Liu et al., 2025). Blood pressure was measured by trained staff using an Omron HEM-7200 device, with participants seated, left arm supported at heart level, and three consecutive readings averaged for accuracy. ePWV was calculated from mean blood pressure (MBP) and age using the following formula: ePWV = 9.587–0.402×age+4.560 × 10^−3^×age^2^–2.621 × 10^−5^×age^2^×MBP +3.176 × 10^−3^×age × MBP –1.832 × 10^−2^×MBP. MBP was derived from systolic (SBP) and diastolic (DBP) blood pressure as: MBP = DBP + 0.4 × (SBP–DBP) (Greve et al., 2016). In addition, ePWV was divided into quartiles to form a ‘quartile categorical variable’ for analysing the risk of developing glaucoma after follow-up (Shi et al., 2024; Ke et al., 2025a; Liu et al., 2025).

Covariates included demographic factors (sex [male/female], marital status [married vs non-married], education [primary school or below, high school, college or above], and location [village vs city/town]); lifestyle factors (smoking status [never/current/former], drinking frequency [never/less than once per month/more than once per month], and sleep duration [hours/day]); and clinical factors (BMI, prior stroke, heart disease, glycemic control, lipid-lowering therapy, and antihypertensive therapy).Glycemic control was defined as the use of hypoglycemic medications or insulin injections, while antihypertensive and lipid-lowering therapies were defined by self-reported medication use.Covariates for the multivariable models were selected *a priori* on the basis of established clinical relevance and prior epidemiological evidence, following the approach described from previous studies (Bozic et al., 2025; Ke et al., 2025a; Ke et al., 2025b).

### Assessment of glaucoma

Glaucoma was assessed through self-reported responses to the CHARLS questionnaire. Participants were classified as having glaucoma if they answered “yes” to the question: “Has a doctor, nurse, or paramedic ever treated you for glaucoma?” Incident glaucoma was determined based on new positive responses during follow-up among participants who were free of glaucoma at baseline (Li et al., 2025).

### Follow-up

The follow-up period was defined as the time elapsing from baseline to either the first self-reported glaucoma diagnosis or the last available follow-up assessment, whichever occurred first. Participants who remained free of glaucoma were censored at the time of their last follow-up interview. Data collection spanned from 2011 (baseline) to 2018 (final follow-up), with a mean follow-up duration of 6.4 ± 1.3 years.

### Statistical analysis

Baseline characteristics across ePWV quartiles were compared using chi-square tests for categorical variables and the Kruskal–Wallis test for continuous variables. Complete case analysis was performed after applying exclusion criteria to remove missing values.

Survival analyses were used to investigate the association between ePWV and glaucoma incidence. Kaplan–Meier survival curves were constructed, and differences across quartiles were tested with the log-rank test. Cox proportional hazards regression models estimated hazard ratios (HRs) and 95% confidence intervals (CIs). Three models were developed: Model 1 (unadjusted); Model 2 (adjusted for sex, marital status, education, location, smoking status, drinking frequency, and sleep duration); and Model 3 (further adjusted for BMI, prior stroke, heart disease, glycemic control, dyslipidemia treatment, and antihypertensive treatment). The proportional hazards assumption was assessed using Schoenfeld residuals, with no significant violations observed.

Restricted cubic spline (RCS) and two-piecewise linear regression models were applied to examine potential nonlinear or threshold relationships between ePWV and glaucoma risk. Subgroup analyses were performed according to demographic and lifestyle factors, with interaction terms included to test for effect modification. Sensitivity analyses were conducted to compare the predictive strength of ePWV against its individual components (age, systolic blood pressure, and diastolic blood pressure).

All analyses were conducted using R software (version 4.3.3). The “survival” package was used for survival analyses, the “rms” package for RCS models, the “segmented” package for threshold analysis, and the “mice” package for multiple imputation when appropriate. A two-sided p-value <0.05 was considered statistically significant. The q-values represent p-values adjusted for multiple comparisons using the false discovery rate (FDR) method.

## Results

### Patient enrollment and follow-ups

**FIGURE 1:**
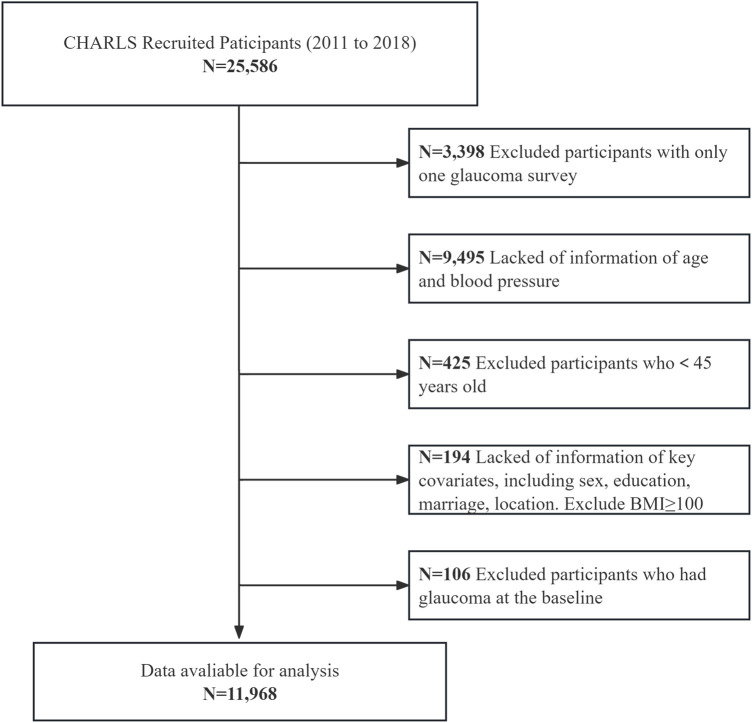
Participant selection flowchart of participant recruitment and exclusion. This resulted in a final analytic cohort of 11,968 participants from CHARLS (2011–2018).

The systematic selection process of study participants from the CHARLS cohort were recruited between 2011 and 2018 (Figure 1). Starting with 25,586 recruited participants, sequential exclusions were applied based on predefined criteria: participants with only one glaucoma survey (n = 3,398), those lacking age and blood pressure information (n = 9,495), participants younger than 45 years (n = 425), those missing key covariates including sex, education, marriage status, location, and participants with BMI≥40 (n = 194), and participants who had glaucoma at baseline (n = 106). The final analytical sample comprised 11,968 participants who met all inclusion criteria and had complete data for the analysis of the association between estimated pulse wave velocity and glaucoma incidence.

### Baseline characteristics of study participants

During 7 years of follow-up, a total of 11,968 participants were stratified into quartiles of estimated pulse wave velocity (ePWV): Q1 (<8.01 m/s), Q2 (8.01–9.14 m/s), Q3 (9.14–10.58 m/s), and Q4 (≥10.58 m/s; n = 2,992 each) (Table 1). Baseline characteristics differed significantly across quartiles (all q < 0.05, FDR-adjusted). Participants in higher ePWV quartiles were older (median age 49 vs 69 years from Q1 to Q4) with progressively higher SBP and DBP, shorter sleep duration, and higher obesity prevalence. The prevalence of glaucoma rose from 2.3% in Q1 to 3.8% in Q4, paralleled by higher rates of stroke (1.1%–3.9%) and heart problems (6.9%–15%). Sociodemographic differences included more males, lower education, and more non-married individuals in higher quartiles. Most participants lived in villages (82%), though city/town residence increased modestly with ePWV. Lifestyle and treatment patterns also reflected greater comorbidity at higher ePWV: ex-smoking was more frequent, regular drinking less common, and use of antihypertensive, lipid-lowering, and diabetes medications substantially higher (all p < 0.001).

**TABLE 1:** Demographics and Baseline characteristics of participants by quartiles of estimated pulse wave velocity (ePWV).

Variable	Overall N = 11,968	Q1 N = 2,992	Q2 N = 2,992	Q3 N = 2,992	Q4 N = 2,992	p-value	q-value^1^
Age, Median (IQR)	58 (51–65)	49 (47–54)	56 (51–59)	60 (56–64)	69 (64–75)	<0.001^2^	<0.001
SBP, Median (IQR)	127.333 (115.000–142.333)	111.333 (105.000–118.000)	124.000 (115.667–132.000)	134.333 (124.333–144.333)	149.667 (137.000–167.333)	<0.001^2^	<0.001
DBP, Median (IQR)	75.000 (67.333–83.667)	67.333 (61.667–72.333)	74.667 (68.667–81.667)	78.667 (71.667–86.333)	82.000 (73.667–91.000)	<0.001^2^	<0.001
Sleep_time, Median (IQR)	6.000 (5.000–8.000)	7.000 (5.000–8.000)	7.000 (5.000–8.000)	6.000 (5.000–8.000)	6.000 (5.000–8.000)	<0.001^2^	<0.001
BMI, Median (IQR)	23.087 (20.806–25.703)	22.754 (20.789–25.047)	23.289 (20.909–25.787)	23.494 (21.080–26.157)	22.905 (20.399–25.730)	<0.001^2^	<0.001
BMI_category, n (%)						<0.001^3^	<0.001
Normal	7,176 (60)	1,959 (65)	1,748 (58)	1,655 (55)	1,814 (61)		
Overweight	3,446 (29)	813 (27)	879 (29)	934 (31)	820 (27)		
Obese	1,346 (11)	220 (7.4)	365 (12)	403 (13)	358 (12)		
glaucoma_status, n (%)						0.008^3^	0.008
No	11,597 (97)	2,923 (98)	2,903 (97)	2,893 (97)	2,878 (96)		
Yes	371 (3.1)	69 (2.3)	89 (3.0)	99 (3.3)	114 (3.8)		
Stroke_status, n (%)						<0.001^3^	<0.001
No	11,688 (98)	2,959 (99)	2,941 (98)	2,914 (97)	2,874 (96)		
Yes	280 (2.3)	33 (1.1)	51 (1.7)	78 (2.6)	118 (3.9)		
Heartproblem_status, n (%)						<0.001^3^	<0.001
No	10,612 (89)	2,787 (93)	2,697 (90)	2,596 (87)	2,532 (85)		
Yes	1,356 (11)	205 (6.9)	295 (9.9)	396 (13)	460 (15)		
SEX, n (%)						<0.001^3^	<0.001
Female	6,290 (53)	1,763 (59)	1,531 (51)	1,466 (49)	1,530 (51)		
Male	5,678 (47)	1,229 (41)	1,461 (49)	1,526 (51)	1,462 (49)		
Education, n (%)						<0.001^3^	<0.001
Primary school or below	8,242 (69)	1,672 (56)	1,912 (64)	2,166 (72)	2,492 (83)		
High school	3,313 (28)	1,230 (41)	986 (33)	714 (24)	383 (13)		
College or above	413 (3.5)	90 (3.0)	94 (3.1)	112 (3.7)	117 (3.9)		
Marital, n (%)						<0.001^3^	<0.001
Non-married	1,472 (12)	147 (4.9)	198 (6.6)	343 (11)	784 (26)		
Married	10,496 (88)	2,845 (95)	2,794 (93)	2,649 (89)	2,208 (74)		
Location, n (%)						<0.001^3^	<0.001
Village	9,788 (82)	2,535 (85)	2,475 (83)	2,409 (81)	2,369 (79)		
City/town	2,180 (18)	457 (15)	517 (17)	583 (19)	623 (21)		
Smoking, n (%)						<0.001^3^	<0.001
Non-smoker	7,241 (61)	1,981 (66)	1,767 (59)	1,736 (58)	1,757 59)		
Current smoker	3,709 (31)	860 (29)	1,011 (34)	947 (32)	891 (30)		
Ex-smoker	1,018 (8.5)	151 (5.0)	214 (7.2)	309 (10)	344 (11)		
Drinking, n (%)						<0.001^3^	<0.001
None of these	8,353 (70)	2,082 (70)	1,990 (67)	2,076 (69)	2,205 (74)		
Drink but less than once a month	1,015 (8.5)	300 (10)	284 (9.5)	236 (7.9)	195 (6.5)		
Drink more than once a month	2,600 (22)	610 (20)	718 (24)	680 (23)	592 (20)		
lipid-lowering therapy, n (%)						<0.001^3^	<0.001
No	11,439 (96)	2,936 (98)	2,847 (95)	2,820 (94)	2,836 (95)		
Yes	529 (4.4)	56 (1.9)	145 (4.8)	172 (5.7)	156 (5.2)		
Glycemic control, n (%)						<0.001^3^	<0.001
No	11,555 (97)	2,933 (98)	2,888 (97)	2,877 (96)	2,857 (95)		
Yes	413 (3.5)	59 (2.0)	104 (3.5)	115 (3.8)	135 (4.5)		
Antihypertensive therapy, n (%)						<0.001^3^	<0.001
No	9,898 (83)	2,880 (96)	2,649 (89)	2,303 (77)	2,066 (69)		<0.001
Yes	2,070 (17)	112 (3.7)	343 (11)	689 (23)	926 (31)		<0.001

Abbreviations: BMI: body mass index, DBP: diastolic blood pressure, IQR: interquartile range, SBP: systolic blood pressure.

^1^False discovery rate correction for multiple testing.

^2^Kruskal–Wallis rank sum test.

^3^Pearson’s Chi-squared test.

### Glaucoma risk across quartiles of estimated pulse wave velocity

**FIGURE 2:**
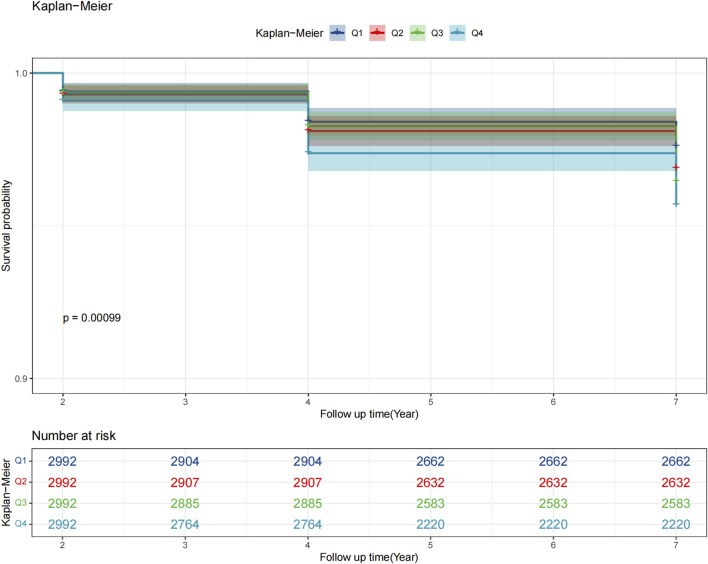
Kaplan–Meier survival curves of glaucoma incidence across quartiles of estimated pulse wave velocity (ePWV). ePWV quartiles (Q1 <8.01 m/s; Q2 8.01–9.14 m/s; Q3 9.14–10.58 m/s; Q4 ≥10.58 m/s).

As shown in Figure 2, Kaplan–Meier curves revealed a stepwise decline in glaucoma-free survival with higher ePWV quartiles, with Q1 showing the most favorable outcomes (log-rank p < 0.05). Cox regression analyses confirmed these findings (Table 2): each 1 m/s increase in ePWV was associated with a 13% higher glaucoma risk in the unadjusted model (HR 1.13, 95% CI 1.07–1.19), and the association remained significant after adjustment for sociodemographic, lifestyle, and clinical factors (HR 1.07, 95% CI 1.01–1.13). Per interquartile increase, risk rose by 36%, 22%, and 19% across models. Quartile analyses showed a dose–response pattern, with Q4 participants having a 39% higher risk compared with Q1 after full adjustment (HR 1.39, 95% CI 1.00–1.93), though the linear trend was attenuated in the fully adjusted model (p for trend = 0.065).

**TABLE 2:** Cox proportional hazards models for glaucoma risk associated with estimated pulse wave velocity (ePWV).

Variable	Model 1			Model 2			Model 3		
	HR	95% CI	p	HR	95% CI	p	HR	95% CI	p
ePWV	1.13	1.07, 1.19	<0.001	1.08	1.02, 1.14	0.006	1.07	1.01, 1.13	0.021
ePWV_per_IVQ	1.36	1.19, 1.55	<0.001	1.22	1.06, 1.41	0.006	1.19	1.03, 1.38	0.021
ePWV_Q									
Q1	Ref	Ref		Ref	Ref		Ref	Ref	
Q2	1.30	0.95, 1.78	0.105	1.26	0.92, 1.73	0.153	1.22	0.89, 1.68	0.211
Q3	1.46	1.07, 1.98	0.016	1.32	0.97, 1.81	0.080	1.27	0.93, 1.75	0.138
Q4	1.81	1.34, 2.44	<0.001	1.49	1.08, 2.05	0.014	1.39	1.00, 1.93	0.049
P for trend			<0.001			0.018			0.065

Abbreviations: ePWV: estimated pulse wave velocity; HR: hazard ratio; CI: confidence interval; IQR: interquartile range; Ref: reference.

Model 1: Unadjusted.

Model 2: Adjusted for sex, marital status, education level, location, smoking status, drinking frequency, and sleep duration.

Model 3: Adjusted for covariates in Model 2, plus BMI, prior stroke, heart disease, glycemic control, dyslipidemia treatment and antihypertensive treatment.

### Threshold effect and dose-response relationship

**FIGURE 3:**
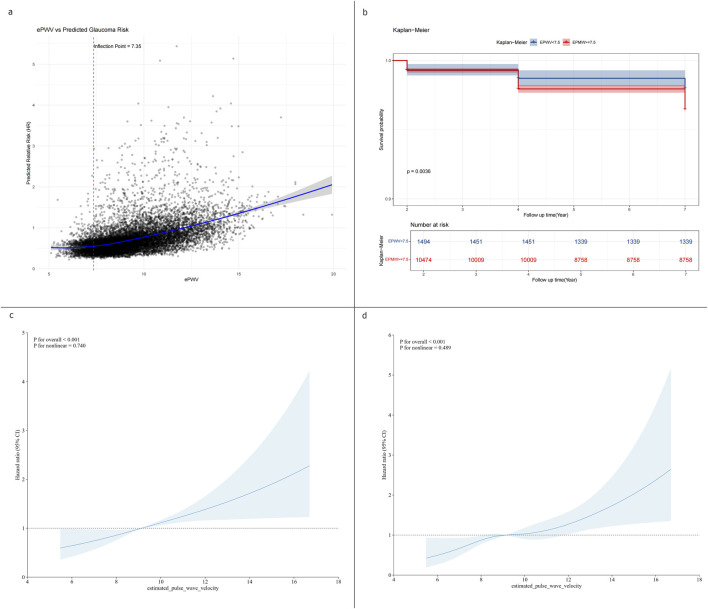
Threshold effect and dose–response relationship between ePWV and glaucoma risk. **(a)** Linear regression model showing a 7% increase in glaucoma risk per 1 m/s increment in ePWV (HR 1.07, 95% CI 1.01–1.13). **(b)** Two-piecewise linear regression model identifying an inflection point at 7.35 m/s **(c)** Restricted cubic spline models showing a steady linear increase in glaucoma risk with higher ePWV. **(d)** Restricted quartic spline models showing a steady linear increase in glaucoma risk with higher ePWV.

Threshold effect analysis was used to assess the dose–response relationship between ePWV and glaucoma risk (Table 3). In the linear model, each 1 m/s increase in ePWV was associated with a 7% higher glaucoma risk (HR 1.07, 95% CI 1.01–1.13, p = 0.021; Figure 3a). A two-piecewise model identified an inflection point at 7.35 m/s, but risks below and above this point were nonsignificant, and model fit was not improved (p = 0.131; Figure 3b). Restricted cubic spline regression showed localized increases at certain knots but no overall evidence of nonlinearity (p = 0.321; Figures 3c,d). Collectively, these results indicate a predominantly linear relationship between higher ePWV and glaucoma risk, without evidence of a threshold effect.

**TABLE 3:** Threshold effect analysis of ePWV on glaucoma risk in the overall cohort.

Outcome	HR	95% CI	p
Model 1 Fitting model by standard linear regression	1.07	1.01, 1.13	0.021
Model 2 Fitting model by two-piecewise linear regression			
Inflection point	7.35		
<7.35	1.74	0.88, 3.43	0.110
≥7.35	1.05	0.99, 1.12	0.121
P for likelihood	0.131		
Model 3 Fitting model by restricted cubic splines linear regression			
1	1.44	0.75, 2.77	0.279
2	13.3	1.32, 134	0.028
3	7.15	1.15, 44.6	0.035
P for likelihood	0.321		

Abbreviations: ePWV: estimated pulse wave velocity; HR: hazard ratio; CI: confidence interval.

### Subgroup analysis

**FIGURE 4:**
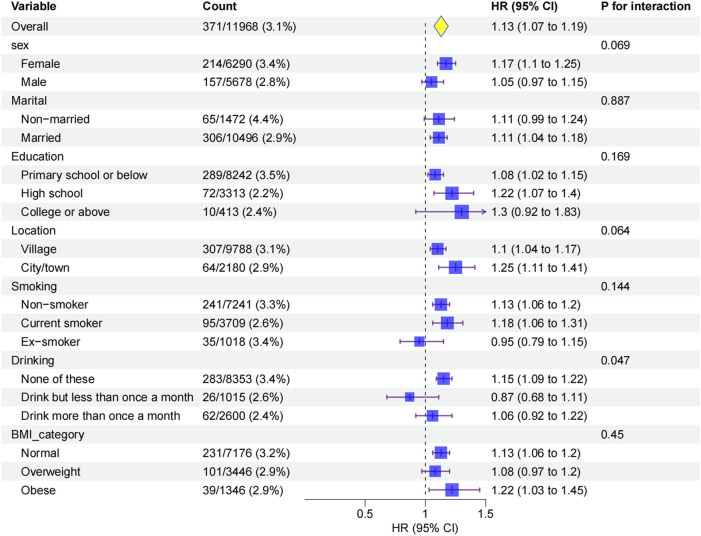
Subgroup analyses of the association between ePWV and glaucoma incidence.Hazard ratios (HRs) and 95% confidence intervals (CIs) were estimated using Cox proportional hazards models adjusted for sociodemographic, lifestyle, and clinical factors (Model 3). Subgroups were stratified by sex, marital status, education, location, smoking status, drinking status, and BMI.

Subgroup analyses showed that the association between ePWV and glaucoma was consistent across sex, marital status, education, residence, smoking, and BMI, with no significant interactions (all P > 0.05; Figure 4). Drinking status was the only significant modifier (P for interaction = 0.047): ePWV was more strongly linked to glaucoma in non-drinkers (HR 1.15, 95% CI 1.09–1.22), whereas no significant association was found in drinkers. Baseline characteristics (Supplementary Table 1) suggested that non-drinkers had higher ePWV, more glaucoma events, and were older, which may partly explain their greater susceptibility. To further clarify whether drinking status independently modified the ePWV-glaucoma association, we performed an age-stratified analysis at the cohort median age of 58 years. In participants aged <58 years, the association between ePWV and glaucoma was not statistically significant (HR 0.95, 95% CI 0.79–1.16,p = 0.628),whereas in those aged ≥58 years the association remained significant (HR 1.07, 95% CI 1.00–1.14,p = 0.049). Importantly, the drinking-status interaction was no longer evident in either age stratum (P for interaction >0.05), indicating that the previously observed stronger association in non-drinkers was not independent of age.These findings indicate that arterial stiffness has a stronger impact on glaucoma among non-drinkers, while the weaker association in drinkers may reflect their younger age profile.

### Sensitive analyses

To validate the independent predictive value of ePWV for glaucoma incidence, we compared its constituent parameters (age and blood pressure) with glaucoma risk (Supplementary Table 2). Age was significantly associated with glaucoma, with each 1-year increase conferring a 4% higher risk (HR 1.04, 95% CI 1.03–1.05, p < 0.001). However, this effect size was smaller than that observed for ePWV (HR 1.13, 95% CI 1.07–1.19), indicating that ePWV exerts a stronger influence on glaucoma incidence than age alone. In contrast, systolic blood pressure (SBP) and diastolic blood pressure (DBP) were not significantly associated with glaucoma risk (SBP: HR 1.00, 95% CI 1.00–1.00, p = 0.977; DBP: HR 0.99, 95% CI 0.98–1.00, p = 0.062), suggesting that blood pressure itself is not an independent risk factor. Overall, these findings underscore that ePWV better captures vascular stiffness status and provides stronger predictive value for glaucoma risk than age or blood pressure alone.

## Discussion

This large prospective cohort study investigated the association between ePWV and incident glaucoma in 11,968 middle-aged and older adults from CHARLS over a 7-year follow-up. We observed a clear stepwise increase in glaucoma incidence across ePWV quartiles, with participants in the highest quartile (≥10.58 m/s) having a 39% higher risk compared with the lowest (<8.01 m/s) after full adjustment. Each 1 m/s increase in ePWV was associated with a 7% increase in glaucoma risk, independent of demographic, lifestyle, and clinical factors. Subgroup analyses showed consistent associations across sex, marital status, education, residence, smoking, and BMI, with drinking status emerging as the only significant modifier: non-drinkers demonstrated a stronger positive association, while no significant relationship was observed among drinkers. Sensitivity analyses further revealed that ePWV had a stronger predictive effect on glaucoma than age or blood pressure alone, underscoring its value as an integrative marker of vascular aging, one with notable potential for AI model development.

Our findings align with recent evidence suggesting that arterial stiffness contributes to glaucoma risk. In the ViDA study, higher carotid-femoral PWV was significantly associated with glaucoma incidence over a decade of follow-up (HR 1.36 per SD increase) (Beros et al., 2024). Cross-sectional studies have also reported that glaucoma patients exhibit higher PWV values and lower macular vessel density, suggesting compromised ocular microcirculation (Lee et al., 2021). Moreover, UK Biobank data demonstrated that elevated pulse pressure, a surrogate of arterial stiffness, was an independent predictor of glaucoma (Macri et al., 2022). Our study extends these findings to a large Chinese cohort, using ePWV as a practical, non-invasive stiffness marker. Notably, while an earlier smaller cross-sectional study found no association, these discrepancies may be explained by limited sample size, design, or population characteristics. Our findings differ from the small cross-sectional study by Chiba’s study in 2008 (Chiba et al., 2008), which reported no association between directly measured Brachial-ankle PWV and prevalent glaucoma in 51 Japanese patients. The difference likely reflects our substantially larger, nationally representative Chinese cohort (n = 11,968), the prospective design capturing incident glaucoma, and the use of validated ePWV derived from age and blood pressure, which together provide greater statistical power and broader generalisability. Taken together, the growing body of evidence supports arterial stiffness as a robust vascular risk factor for glaucoma across populations, reinforcing ePWV’s relevance for data-driven AI applications.

The biological plausibility of our findings rests on the vascular theory of glaucoma. Arterial stiffening impairs the Windkessel effect of large arteries, increasing pulse pressure and reducing diastolic perfusion, thereby destabilizing ocular blood flow and compromising optic nerve head autoregulation (Choi and Kook, 2015). This hemodynamic stress may predispose to ischemia-reperfusion injury of retinal ganglion cells. Additionally, arterial stiffness reflects cumulative vascular aging, endothelial dysfunction, and microvascular remodeling, all of which can reduce optic nerve perfusion reserve (Beros et al., 2024). The stronger association in non-drinkers could be partly explained by their older age and higher baseline stiffness, while younger drinkers may not yet manifest stiffness-related glaucoma risk (Del Giorno et al., 2022). The stronger association originally observed in non-drinkers is largely explained by their older age and higher baseline stiffness. Age-stratified analyses confirmed that the ePWV-glaucoma association remained significant only in participants aged ≥58 years, and the drinking-status interaction disappeared (P for interaction >0.05). Chronic alcohol consumption has complex vascular effects—moderate intake may transiently enhance endothelial function, whereas heavy or long-term use is associated with increased arterial stiffness and impaired vascular compliance (Del Giorno et al., 2022; Schutte et al., 2024; Gusti et al., 2025). These mechanisms could partly influence the observed interaction; however, this result should be considered hypothesis-generating and requires confirmation in cohorts with detailed alcohol-consumption data.

In normal tension glaucoma, higher carotid femoral pulse wave velocity (PWV) has been correlated with lower macular vessel density measured by OCTA(Lee et al., 2021).Systemic blood pressure profiles related to arterial stiffness also influence ocular perfusion pressure (OPP), a recognized risk factor for open angle glaucoma (Kim et al., 2020). Because estimated PWV (ePWV) is a validated surrogate for carotid femoral PWV(Greve et al., 2016), elevated ePWV likely reflects the same hemodynamic burden that lowers OPP and compromises retinal capillary density, providing a biologically plausible pathway from systemic stiffness to glaucomatous neurodegeneration (Wang et al., 2023). Overall, these mechanisms highlight the role of systemic vascular health in glaucomatous neurodegeneration beyond intraocular pressure-strengthening the rationale for incorporating ePWV into AI models that aim to capture multi-dimensional glaucoma risk.

Our study provides evidence that ePWV, a simple and non-invasive marker derived from age and blood pressure, may serve as an independent predictor of glaucoma risk.

The marginal p for trend (0.065) after full adjustment likely reflects confounding by antihypertensive therapy (included in Model 3).These medications reduce arterial stiffness while potentially modifying glaucoma risk. Nevertheless, the significant HR for Q4 vs Q1 (1.39, 95% CI 1.00–1.93) still support a meaningful dose-response relationship, with the marginal trend attributed to residual variability from covariate adjustment. As glaucoma often remains asymptomatic until advanced stages, incorporating vascular markers such as ePWV into risk stratification could improve early identification of high-risk individuals (Beros et al., 2024). From a preventive perspective, interventions to mitigate arterial stiffness, which can be conducted through lifestyle modifications or pharmacologic therapies, may hold promise in reducing glaucoma burden, particularly in aging societies like China. Our observation that each 1 m/s increase in ePWV was associated with a 7% higher risk of glaucoma, supporting biological plausibility for a stiffness-related pathway (Lee et al., 2022; Beros et al., 2024). Moreover, incident-glaucoma analyses using arterial stiffness measures show prospective associations (Lee et al., 2022; Beros et al., 2024).ePWV reflects cumulative arterial stiffening (Greve et al., 2016), supplying additional systemic information beyond ocular imaging. Integrating vascular health monitoring into ophthalmic practice could thus represent a novel approach to comprehensive glaucoma prevention and management.

Several limitations should be acknowledged. First, glaucoma was self-reported without confirmatory optic-nerve imaging (fundus photography or OCT) or IOP and misclassification cannot be excluded (Paul et al., 2023; Higgins et al., 2024). Large epidemiologic and Mendelian-randomization analyses indicate only small positive effects of systemic blood pressure on IOP(Plotnikov et al., 2022). In our models, adjustment for blood pressure and antihypertensive therapy should reduce confounding through IOP. Second, intraocular pressure and detailed ophthalmic examinations were unavailable, precluding subtype analyses, e.g., normal-tension vs. high-tension glaucoma that could refine AI model stratification.Future studies should incorporate these indicators to enable more precise risk stratification in AI-based screening models.Third, although ePWV is validated, it is an indirect surrogate and not a direct measure of carotid-femoral PWV, which may affect its predictive precision in AI systems. This indirect derivation from age and blood pressure may introduce nondifferential measurement error that could attenuate effect sizes and slightly reduce the incremental value of ePWV in multimodal AI models (Alansare et al., 2022; Heffernan et al., 2023). Future studies comparing AI models that incorporate ePWV with those using directly measured cfPWV will be valuable to confirm robustness and reliability.Fourth, residual confounding from unmeasured factors, e.g., family history, ocular perfusion pressure, cannot be ruled out. Finally, our findings are based on Chinese adults and may not be generalizable to other populations, emphasizing the need for diverse datasets to optimize AI model generalizability. Our cohort consisted of older Chinese adults, and 18% of the analytic sample lived in urban areas.Future studies in younger cohorts and in more highly urbanised populations are needed. Future studies integrating direct vascular measures, comprehensive ophthalmic data, and interventional designs are warranted to confirm causality and explore clinical translation, including the development of ePWV-integrated AI screening tools.

Notably, unlike other vascular markers such as carotid intima-media thickness, which requires ultrasound or pulse pressure, which is more sensitive to short-term blood pressure fluctuations, ePWV only needs age and routine blood pressure for calculation (Greve et al., 2016; Bozic et al., 2025). Both are readily available in primary care at low cost, avoiding specialized equipment, reducing screening costs, and enabling seamless integration into AI models using common health records (Cheng et al., 2024). Beyond single-modality AI, ePWV could also be combined with ocular imaging data such as fundus photographs or OCT in multimodal AI systems to enhance glaucoma risk prediction and provide complementary systemic vascular information, adding it may help in cases where ocular imaging is equivocal, e.g., optic disc margin ambiguity and high myopia (Huang et al., 2023; Heckel et al., 2025; Koornwinder et al., 2025; Sivakumar and Penkova, 2025). This enables ePWV to be computed automatically from routine blood-pressure data and to flag high-risk individuals for targeted glaucoma screening in community and primary-care settings.

## Conclusion

In summary, this nationally representative cohort study demonstrates that higher ePWV is independently associated with increased glaucoma risk in middle-aged and older Chinese adults. The relationship was linear, consistent across most subgroups, and stronger in non-drinkers. Compared with age or blood pressure alone, ePWV provided superior predictive value, suggesting it captures cumulative vascular aging relevant to glaucomatous neurodegeneration. These results suggest that ePWV could serve as a promising candidate marker for incorporation into future AI-assisted glaucoma screening strategies in middle-aged and older Chinese populations.

## Data Availability

Publicly available datasets were analyzed in this study. This data can be found here: https://charls.charlsdata.com/users/profile/index/zh-cn.html. The CHARLS protocol was approved by the Biomedical Ethics Review Committee of Peking University (IRB00001052-11015) and conducted in accordance with the Declaration of Helsinki. Written informed consent was obtained from all participants. This study adheres to the Strengthening the Reporting of Observational Studies in Epidemiology (STROBE) guidelines.

## Abbreviations

CHARLS, China Health and Retirement Longitudinal Study; ePWV, estimated pulse wave velocity; IOP, intraocular pressure measurement; OCT, optical coherence tomography; OCTA, optical coherence tomography angiography; OPP, ocular perfusion pressure; PWV, pulse wave velocity.
