# Supplementary material for: ePWV as a scalable risk factor for large-scale glaucoma screening: evidence from a national Chinese cohort
Source: Front Cell Dev Biol. 2025 Oct 27;13:1700378. doi: 10.3389/fcell.2025.1700378 (PMC12598034; doi:10.3389/fcell.2025.1700378)
Supplement: Supplementary file 1 [file Table1.docx]

| Supplementary Table1. Baseline characteristics of study participants stratified by drinking status | | | | | |
| --- | --- | --- | --- | --- | --- |
| Variable | Overall  N = 11,968^1^ | None of these  N = 8,353^1^ | Drink but less than once a month  N = 1,015^1^ | Drink more than once a month  N = 2,600^1^ | p-value^2^ |
| ePWV | 9.14 (8.01, 10.58) | 9.20 (8.02, 10.68) | 8.79 (7.85, 10.17) | 9.08 (8.07, 10.45) | <0.001 |
| Age | 58 (51, 65) | 58 (52, 65) | 56 (50, 63) | 57 (51, 63) | <0.001 |
| Glaucoma happened | 371 (3.1%) | 283 (3.4%) | 26 (2.6%) | 62 (2.4%) | 0.021 |
| Abbreviations: ePWV: estimated pulse wave velocity;  ^1^Median (Q1, Q3); n (%)  ^2^Kruskal-Wallis rank sum test; Pearson's Chi-squared test | | | | | |
