# Supplementary material for: ePWV as a scalable risk factor for large-scale glaucoma screening: evidence from a national Chinese cohort
Source: Front Cell Dev Biol. 2025 Oct 27;13:1700378. doi: 10.3389/fcell.2025.1700378 (PMC12598034; doi:10.3389/fcell.2025.1700378)
Supplement: Supplementary file 2 [file Table2.docx]

| Supplementary Table2. Cox proportional hazards models for glaucoma risk associated with component parameters of ePWV | | | | | | | | | |
| --- | --- | --- | --- | --- | --- | --- | --- | --- | --- |
| Variable | Model 1 | | | Model 2 | | | Model 3 | | |
|  | HR | 95% CI | p | HR | 95% CI | p | HR | 95% CI | p |
| Age | 1.04 | 1.03, 1.05 | <0.001 |  |  |  |  |  |  |
| SBP |  |  |  | 1.00 | 1.00, 1.00 | 0.977 |  |  |  |
| DBP |  |  |  |  |  |  | 0.99 | 0.98, 1.00 | 0.062 |
| Abbreviations: ePWV: estimated pulse wave velocity; DBP:Diastolic Blood Pressure; SBP:Systolic Blood Pressure;HR: hazard ratio; CI: confidence interval; | | | | | | | | | |
